# Nutrition knowledge, attitudes and practices of primary school children in Tshwane Metropole, South Africa

**DOI:** 10.4102/phcfm.v11i1.1846

**Published:** 2019-04-30

**Authors:** Nomsa P.S. Mamba, Lizeka Napoles, Nelly M. Mwaka

**Affiliations:** 1 School of Health Systems and Public Health, University of Pretoria, Gezina, Pretoria, South Africa

**Keywords:** nutrition knowledge, nutrition attitudes, nutrition practices, nutrition interventions, primary school learners, South Africa

## Abstract

**Background:**

The increasing prevalence of being overweight and obesity in South African school children requires interventions that are evidence based. Nutrition knowledge, attitudes and practices (KAP) studies are thus needed to provide evidence for the planning of interventions that address and prevent nutrition problems in school children.

**Aim:**

The aim of the study on which this article is based was to assess nutrition knowledge, attitudes and practices of grade 4–6 learners from three primary schools in a South African township. The article seeks to highlight the key results of this quantitative study.

**Setting:**

The study took place in three primary schools in Mamelodi township, Pretoria, South Africa.

**Methods:**

Data were collected from grade 4–6 learners using self-administered questionnaires. After coding and collating data using Epi info^TM^, STATA was then used for analysis. A description of KAP results was carried out using simple descriptive statistics, while the associations were tested using a chi-square test.

**Results:**

Learners displayed inadequate knowledge of a balanced diet (23%) as well as low knowledge of food groups. With regard to attitudes, the most liked food group was the drinks and snacks (72.9%), while the least liked food group was the fruits and vegetables (8.11%). With regard to practices, the most frequently consumed food group was the drinks and snacks (72.6%), while fruits and vegetables were the least consumed. However, 78.91% of the learners displayed very good nutrition-related practices, such as making their own breakfast and eating breakfast.

**Conclusion:**

The inadequate knowledge displayed by learners indicates a gap with nutrition education in the curriculum. There is a need to explore innovative and novel approaches to improve nutrition knowledge of school children. Parents also need to be targeted to ensure better outcomes.

## Background

Children of primary school age are responsive to health messages and behaviour changes which may be maintained into adolescence and adulthood.^1,2^ For this reason, nutrition interventions targeted at this age group are most likely to have long-term positive effects such as improved nutrition-related practices; reduced nutrition-related problems such as obesity, being overweight, under-nutrition; and nutrition-related chronic diseases. It is essential to assess children’s own nutrition knowledge, attitudes and practices (KAP) in order to plan meaningful nutrition interventions that will address gaps as well as factors that influence these aspects.

The development of good nutrition practices is influenced by a number of factors and the knowledge–Attitude–Behaviour (KAB) model provides a framework for the change in nutrition practices. This model suggests that knowledge is a prerequisite for behaviour change. As knowledge increases, attitudes begin to change and, over time, behaviour changes.^3^

An increasing prevalence of overweight and obese children in primary schools has been observed worldwide, including South Africa. Although it is highly prevalent in children from urban areas, this is also an increasing trend in children from the rural areas.^4,5,6,7,8^ According to Reddy et al., the national prevalence of overweight children is at 17% and obesity is at 5% among South African school children.^8,9,10^ Several factors contribute to being overweight and obese including dietary intake, urbanisation, industrialisation and socioeconomic status of the family. In most cases, dietary intake is directly linked to being overweight and obese among school children^7,11,12^ and, according to Sahota et al., dietary intake is greatly influenced by nutrition KAP.^7^ Steyn states that poor dietary behaviour, referring to high intake of unhealthy snacks, is a high risk factor for the development of being overweight and obese in South African school children.^12^

On the other hand, under-nutrition is also prevalent in some parts of South Africa. Stunting is the most prevalent form of under-nutrition, followed by being underweight and then wasting. The national prevalence of stunting among school children is at 13%; underweight at 8% and wasting at 4%.^1,9,10^ While poverty is directly associated with stunting in South Africa,^13^ it has also been reported that parents’ inadequate nutrition knowledge, children’s poor nutrition practices and infections contribute to the rate of stunting.^7^

Globally, very little has been published to demonstrate children’s own level of nutrition KAP. The focus has been on the knowledge of care givers as well as parents that are responsible for purchasing food and feeding children.^14^ A few studies have assessed knowledge and attitudes only^15^ or practices with regard to specific food groups.^16^ In South Africa, one study assessed nutrition knowledge only^17^ and the other nutrition practices only.^18^ Both studies were localised in selected regions of South Africa and cannot be generalised for all other regions, owing to variations in culture.

The assessment of the learners’ own nutrition KAP helps the public nutrition practitioners plan appropriate nutrition interventions, to reduce the impact of the stated nutrition problems. The South African government has developed an Integrated School Health Policy (ISHP) which incorporates nutrition education. Within the school health programme, the learners’ level of nutrition knowledge as well as their nutrition-related attitudes and practices can be addressed.^19^

Schools provide a suitable setting for influencing children’s health and health behaviour and primary schools are particularly suitable for nutrition behaviour change programmes.^1,2,12^

## Methods

### Study design

The study on which this article is based was a cross-sectional descriptive study.

### Setting

The study took place in three primary schools in Mamelodi township, Pretoria, South Africa. The three participating schools form part of the Health Promoting Schools pilot programme facilitated by the University of Pretoria’s School of Public Health.

### Participants and sampling strategy

The study population was composed of all learners in grades 4–6 from three primary schools in Mamelodi township of South Africa. A total sample size of 310 learners was obtained from all three schools with a total enrolment of 864, and consisted of all the learners who returned consent forms signed by their parents. A total of 12 questionnaires were disqualified owing to too much missing information, bringing the total number of questionnaires that were analysed to 298.

### Data collection

A standardised nutrition KAP questionnaire was adopted from the HealthKick programme in South Africa with modifications. The instrument contained simple multiple choice questions with colourful pictures. The instrument was pretested in a primary school within the same location and with a similar setting to those of the target schools. The pretest was to observe factors such as time, the understanding of questions and it was then corrected for errors. The instrument was modified following the findings of the pretest. With regard to nutrition knowledge, learners were asked to identify a picture of a balanced diet and match food groups with their functions in the body. Regarding attitudes, learners were asked to rank their liking of popular foods on a scale of 1 to 5, where 1 was the least liked and five the most liked. For nutrition practices, learners were asked to rank the consumption of the same foods on a scale of 1 to 5 within the last week of data collection, where 1 was not consumed at all in the last week and 5 consumed more than five times in the last week. In addition, nutrition-related practices were determined by indicating the learners’ ability to prepare and eat breakfast, pack their lunch box, their food choices and involvement in physical activities.

### Data collection procedure

Consent was obtained from the three schools, the provincial Department of Education and University of Pretoria Ethics Committee, following successful submission of the protocol. The target population which consisted of learners in grades 4–6 from the three primary schools were all given consent forms to take to their parents. Only learners who returned their signed consent form took part in the study, making a total of 310 learners. The participating learners were then administered with an assent form prior to responding to the questionnaire, which took about 30–45 min to complete. A standardised KAP questionnaire was self-administered by the learners in the presence of a researcher, a teacher and the local health promoter. The researcher explained the instrument to the learners and where clarification was needed, the teacher and health promoter helped.

The data collection process was conducted over a period of 3 days at the three schools. The principal introduced the research and the researcher to the participating learners who were assembled in one classroom after school.

The collected data were coded, then entered into Epi info^TM^ with 12 disqualified entries, and then analysed.

### Data analysis

Data analysis was conducted using STATA. Simple descriptive statistics were used to describe learners’ KAP. For the food list used to assess learners’ attitudes, new variables were generated to reduce the food list. The generated variables were meant to group the foods into cereals, proteins, fruits, vegetables, drinks and snacks.

### Ethical considerations

Approval for the study was obtained from the Ethics Committee of the University of Pretoria (426/2013). Parents who signed the consent form allowed their children to form part of the study; learners who took part in the study also signed a consent form prior to answering the questionnaire. Permission was also sought from the Gauteng Department of Education as well as from the authorities of the three schools.

## Results

### Learners’ demographic information

Learners from three primary schools in Mamelodi township, South Africa, participated in the study. Learners’ demographic information was limited to the school, the learners’ gender, his or her age and the grade level. Of the total number of participants, 191 were girls and 105 were boys. The highest number of learners (150) were in the middle age group (11–13 years), followed by the lower age group (8–10 years) where there were 141 participants. The age group with the lowest number of participants was the 14–15 years age group where only five participants took part in the study. Regarding the grade levels, 111 learners participated from grade 4, followed by 106 from Grade 6 and 78 from Grade 5.

### Description of learners’ nutrition knowledge

Learners were found to have inadequate nutrition knowledge. Only 23.2% were able to identify picture C, which showed a balanced diet. Regarding the food groups, the most known food group to learners was the body building category with 45.3% correctly identifying this food group, while the least identified food group was the fibre group at 20%. In most cases, learners did not understand the term fibre. Table 2 shows the percentage of the learners who could correctly identify a balanced diet. *Only 69 out of 298 learners (23.2%) were able to identify the picture which showed a balanced diet.*

**TABLE 1:** Learners’ demographic information, as captured on the questionnaire.

Schools	Frequencies per variable							
	Sex (%)		Age group (%)			Grade level (%)		
	Boys (*N* = 105)	Girls (*N* = 191)	8–10 (*N* = 141)	11–13 (*N* = 150)	14–15 (*N* = 5)	Grade 4 (*N* = 111)	Grade 5 (*N* = 78)	Grade 6 (*N* = 106)
Mogale Primary School	31.4	5.6	39.7	30	0	26.2	47.4	33
Pula Difate Primary School	35.3	35.1	35.5	35.3	20	36	37.2	32.1
Rethakgetse Primary School	33.3	29.3	24.8	34.7	80	37.8	15.4	34.9

**TABLE 2:** Identification of a balanced diet (*N* = 298).

Balance diet	Frequency	Percentage
B: some food groups	152	51
A: all food groups	74	24.8
C: all food groups	69	23.2
D: none of the above	3	1

### Description of learners’ attitudes towards nutrition

When learners were asked to identify foods which they liked, for cereals it was bread with 56.9%, for proteins it was yoghurt with 54.4%, for fruits and vegetables it was oranges with 66.1% and for drinks and snacks it was biscuits with 72.9%. In general, drinks and snacks was the most liked food group. Table 3 shows the most/least liked foods as well as most/least consumed foods. *The most liked foods are the drinks and snacks.*

**TABLE 3:** Learners’ nutrition attitudes.

Food items	Like	
	Frequency	Percentage
**Cereals**		
Bread	169	56.9
Rice	154	51.9
Porridge	122	41.1
Sweet potato	71	24.0
Samp	54	18.3
Cassava	37	12.5
**Proteins**		
Yoghurt	161	54.4
Milk	160	54.1
Chicken	159	53.9
Fish	137	46.4
Cheese	119	40.2
Peanuts	108	36.5
Beef	107	36.2
Eggs	103	34.9
Bean	73	24.7
Jugo beans	50	16.9
**Fruits and vegetables**		
Orange	195	66.1
Banana	186	62.8
Apple	177	60.0
Carrots	54	18.3
Cabbage	36	12.2
Onions	35	11.8
Spinach	28	9.46
Tomatoes	24	8.11
**Drinks and snacks**		
Biscuits	210	72.9
Soft drinks	209	70.9
Cakes	202	68.2
Sweets	182	61.7
Chocolates	166	56.1
Chips	145	49.2
Juice	119	40.2
Water	76	25.7

### Description of learners’ nutrition practices

Regarding learners’ frequent consumption of the same food items; for cereals, bread was the most consumed with 56.3% and cassava was the least consumed, although in most cases learners did not know what cassava was. The most consumed protein within the last week of data collection was chicken with 50.5%, plant proteins were the least consumed form of protein and some foods such as jugo beans were not known to most learners. The most consumed fruit was an apple with 69.6% yet the most liked was the orange perhaps owing to the fact that oranges are seasonal fruits. Vegetables were the least consumed food group as a whole. The highest consumed vegetable was onion with 18%, perhaps owing to its regular use during cooking. Drinks and snacks were the most highly consumed food group by learners with sweets scoring 72.6% as learners had consumed sweets more than five times in the last week of the research. Table 4 shows the most/least consumed foods. *The most consumed foods are the drinks and snacks.*

**TABLE 4:** Learners’ nutrition practices.

Food items	Consumed	
	Frequency	Percentage
**Cereals**		
Bread	166	56.3
Rice	94	31.9
Porridge	83	28.1
Samp	79	26.6
Sweet potato	58	19.7
Cassava	26	8.8
**Proteins**		
Chicken	149	50.5
Milk	142	48.1
Eggs	139	47.0
Yoghurt	125	42.4
Fish	98	33.2
Cheese	87	29.3
Beef	80	27.1
Peanuts	74	25.1
Bean	63	21.4
Jugo beans	30	10.2
**Fruits and vegetables**		
Apple	206	69.6
Orange	202	68.9
Banana	160	54.2
Onions	53	18.0
Carrots	51	17.4
Tomatoes	44	14.9
Cabbage	32	10.9
Spinach	31	10.5
**Drinks and snacks**		
Sweets	215	72.6
Biscuits	205	68.8
Chips	177	59.8
Chocolates	135	45.8
Cakes	128	43.4
Juice	123	41.7
Water	115	39.0
Soft drinks	105	35.6

### Description of learners’ nutrition-related practices

A total of 79.9% of learners said they do have school lessons on nutrition, while a good percentage (78.9%) do eat breakfast and they are able to make their own breakfast (72.7%). Although 57.8% said they can make their lunch box, only 41.8% carry a lunch box to school, this might be because 69.4% take money to school which they use to buy sweets and snacks.

Learners were further assessed for good choices of fat and 28.8% of learners said they would not choose meat with skin as a good source of fat, 18.5% said they would not choose chips and only 29.5% said they would choose nuts as a good source of fat. Sixty-four per cent said they would choose soft margarine as fat, 51.5% said they would take avocado as a good source of fat, 31.9% said they would not take doughnuts as their source of fat and 34.3% said they would not take pilchards as their source of fat. Table 5 shows the percentage of learners who indicated having a particular nutrition-related practice. *Learners displayed good nutrition-related practices.*

**TABLE 5:** Learners’ nutrition-related practices.

Nutrition practice	Frequency	Percentage
Have school lessons on nutrition	235	79.9
Eat breakfast	232	78.9
Make breakfast	214	72.8
Take money to school	204	69.4
Make lunch box	170	57.8
Choose what to eat	134	45.6
Carry a lunch box	123	41.8

Furthermore, learners were asked to identify physical activity that they do and the following were the results:

### Description of learners’ physical activity

A total of 87.5% regard playing games as physical activity, 79.7% said they have fun when doing physical activity, 72.9% said they did get encouragement from teachers to do physical activities, while 62.7% said they also get encouragement from family members; 83.1% said they do have organised sporting activities in their schools with 72.9% saying they take part in those sporting activities. A total of 38.6% of learners said they do not like sports. When learners were asked how much time they spend watching TV or playing TV/computer games a day, 44.4% said they spend over 1–2 hours per day, 24.8% said they spend 30–60 minutes per day and 30.9% said they spend less than 30 min a day. This is to say that most of the learners spend most of their after school hours watching TV or playing TV/computer games. On the other hand, learners regard engaging in organised sporting activity as the best for their health and 44.1% said they liked organised sporting activities, yet only 32.9% said they play outdoor games the most than any other form of physical activity. Table 6 shows the percentage of learners who engage in a particular physical activity. *Learners displayed good physical activity although 38.6% said they do not like sports.*

**TABLE 6:** Appropriate physical activity.

Physical activity	Frequency	Percentage
Regard playing games as physical activity	258	87.5
Have organised sporting activity at school	245	83.1
Have fun in physical activity	235	79.7
Encouragement by teacher to do physical activity	215	72.9
Take part in sports at school	215	72.9
Encouragement by family to do physical activity	185	62.7
Do not like sports	114	38.6

## Discussion

This study assessed the nutrition KAP of grade 4–6 learners from three primary schools in a South African township. In this study, learners’ nutrition knowledge was described based on their ability to identify the correct picture which showed a balanced diet and to link the pictures of food groups with their functions. The assessment indicates limited nutrition knowledge. This is consistent with a study conducted by Vijayapushpan et al. in Hyderabad which identified economic transition and urbanisation to be the leading cause of nutrition transition from an African diet to the western diet, and this is attributed to lack of nutrition knowledge.^20^

Vijayapushpan et al.^20^ describe the African diet as one that originated from plant sources and had lots of fibre; in this study, the food group with foods based on plant sources got very low scores. While bread and common fruits like apples, bananas and oranges got high scores, these were significantly lower than the food group comprising sweets, chips and biscuits. This indicates that the most consumed foods were refined foods with very low fibre content.

Oldewage-Theron and Egal^17^ conducted a study to determine the nutrition knowledge and the nutrition status of primary school children in QwaQwa, a rural town in South Africa. They found that most children had an average knowledge about basic nutrition, although a gap was identified in the role of various food groups in the diet. This same study also identified a gap in learners’ knowledge of the functions of food groups in the body and further found that learners have inadequate nutrition knowledge regardless of the high indication of school lessons obtained in nutrition education.^17^

The result of our study on nutrition knowledge is also consistent with the results of a study conducted by Koo et al. on KAP of primary school children towards grains. They found that 70.3% of children had a low knowledge of the grains.^16^ Vereecken relates children’s limited nutrition knowledge to their mothers; she argues that children can only get to know what their mothers give them.^21^

Harris^22^ studied the role of attitudes and barriers on the implementation of a nutrition intervention in primary school children and found that nutrition knowledge depends on the child’s age and type of school education and that there was no difference in nutrition knowledge of obese and non-obese children. This was confirmed in a study conducted by Reinehr.^23,^

Secondary findings of our study found that the learners’ ability to identify a food group that provides the body with the best energy and a food group that contains proteins is associated with the learners’ age. Our study, however, did not look at the type of nutrition education offered to learners and a recommendation in this regard is to be made for further studies.

With regard to nutrition attitudes and practices, our study found that the most liked and frequently consumed food group was ‘drinks and snacks’ where the learners demonstrated frequent consumption of unhealthy snacks. Apart from the fact that children like sweets, this practice might be perpetuated by the type of food items sold by the vendors at the school gates which was observed during data collection visits. Shariff et al.^24^ and Piscopo^25^ note that food consumption is associated with foods that are available and accessible in the children’s environment. The habit of not making a lunch box and carrying money to school could be a contributing factor, as the money learners carry is mainly used to buy energy-dense snacks. This is confirmed in a study conducted by Temple et al. in the Western Cape, South Africa, where they found that children who do not carry a lunch box to school tend to buy unhealthy energy-dense snacks from the shops and vendors around the schools.^26^

With regard to nutrition-related practices, the study found that the majority of learners could make their own breakfast and also had breakfast. A similar study conducted by Lin et al. with elementary children found that only 25% of these children ate breakfast even though they could make their own breakfast.^3^ The lunch box practice however was contrary to the findings of the study conducted by Abrahams et al. in the Western Cape Province of South Africa, where they found that about 70% of learners carried lunch boxes to school. The majority of learners in both cases indicated they could prepare their own lunch boxes.^3,18^

Of great interest, however, are the results of our study with regard to food choices, where the majority of learners indicated they would choose ‘good’ fat sources like nuts and avocado over ‘bad’ fat sources like chicken skin and potato crisps. This could have been a result of emphasis on choice of fats in the curriculum as well as the approach used. Worsley observes that when procedural knowledge is acquired, it can result in behaviour change.^27^

With regard to physical activity, our study found that a high percentage of learners spend over 1–2 h a day watching TV or playing TV/computer games. These results confirm the findings of Triches^28^ and Naidoo^29^ who both agreed that 25% of children in South Africa spend more than 3 h in sedentary behaviour which is a high risk factor for nutrition-related health problems.^28,29,30^

### Limitations

The main limitation of the study is that the curriculum was not examined before the questionnaire was finalised, to determine the current nutrition content, the type of nutrition education covered and the approaches used for the delivery of nutrition messages.

## Conclusion

The main aim of this research was to assess the nutrition KPA of learners in South African township primary schools. The findings of the study indicate that there is a gap in the learners’ nutrition knowledge, especially of the balanced diet and food groups, and this negatively impacts their nutrition attitudes and practices. Learners generally like and consume more energy-dense and less nutritious foods, while their liking for, and consumption of, vegetables is low. The study confirms the theory of the KAB model that nutrition knowledge is a prerequisite for behaviour change. In this case, limited nutrition knowledge, especially of a balanced diet and the food groups, results in increased liking and consumption of the more energy-dense and less nutrient-dense foods.

The study further demonstrates that the learners’ practices are influenced by their exposure to certain foods; learners generally eat what is available at home or at school. The recommendation therefore is to increase learners’ exposure to more healthy foods like fruits and vegetables than unhealthy snacks. The exposure should be a joint effort by the school to improve the school environment and homes by providing these. Interventions to regulate foodstuffs that are sold by vendors at schools must be explored as well as those that promote the carrying of lunch boxes by learners from home.

The study highlights the need to strengthen nutrition education in schools, in particular the approaches for its delivery methods. The delivery methods employed to teach nutrition must be appropriate for the impact needed on KAP of the learners. Nutrition education in the curriculum should explore more innovative and novel approaches to improve nutrition knowledge. This could use gamification as one of the methods used to teach certain aspects of nutrition as well as the use of school vegetable gardens as teaching tools.

Interventions should target improving learners’ nutrition knowledge which will have a positive impact on their attitudes as well as their practices. Some interventions must target the parents of learners, to play a major role in shaping their children’s nutrition KAP at an early age such that they grow with it into adulthood and reduce the risk of non-communicable diseases. School health policies should be enforced to improve the school environment and make it favourable for learners to make good food choices while at school. The nutrition education curriculum should also be looked at as well, as a mode of delivery.
